# Diagnostic buccal d´une carence en vitamine B12 due à un traitement au long court avec le metformine: à propos d´un cas

**DOI:** 10.11604/pamj.2020.37.280.19776

**Published:** 2020-11-26

**Authors:** Nourdine Attiya

**Affiliations:** 1 Environnement et Santé, Département de Biologie, Faculté des Sciences et Techniques d’Errachidia, Université Moulay Ismaïl de Meknès, Meknès, Maroc

**Keywords:** Metformine, vitamine B12, glossite de Hunter, stomatodynie, *case report*, Metformin, vitamin B12, Hunter's glossitis, stomatodynia, case report

## Abstract

Le metformine est actuellement placé en première ligne pour le traitement du diabète de type 2. Il est également indiqué dans le diabète de type 1 en cas de résistance à l´insuline. Il présente de nombreux effets bénéfiques sur le métabolisme des carbohydrates, la perte de poids et la protection vasculaire. Cependant, il peut également engendrer d´importants effets indésirables comme le risque d´anémie en cas d´utilisation prolongée. Il est rapporté que les patients sous metformine au long court présentent une réduction de la concentration sérique de la vitamine B12. Les signes buccaux associant une glossite de Hunter et une stomatodynie peuvent être révélateurs et conduire à un diagnostic précoce d´une carence en vitamine B12. Nous rapportons ici le cas d´une patiente qui présente ces signes buccaux depuis 2 ans et dont les bilans biologiques ont révélé une anémie normocytaire ferriprive avec carence en vitamine B12. La prise prolongée du metformine a été considérée comme étiologie de l´avitaminose B12 en attendant d´écarter d´autres causes probables. La correction par injection intramusculaire d´hydroxocobalamine avec du fer par voie orale ont permis la normalisation des taux sériques et la disparition des signes cliniques. La patiente a été adressée à un centre spécialisé pour un bilan étiologique plus poussé. Ce cas clinique met en relief le rôle important que peut jouer le médecin dentiste dans le diagnostic précoce de la déficience en vitamine B12 et la prévention de son évolution qui peut être dramatique en cas de découverte tardive.

## Abstract (en)

Metformin is the first line treatment for type 2 diabetes. It is also indicated in patients with insulin-resistant type 1 diabetes. It has several benefic effects on carbohydrate metabolism, weight loss and vascular protection. However, it can also cause serious adverse reactions such as the risk of anemia associated with long term use. It has been reported that long-term metformin use might reduce serum vitamin B12 levels. Oral signs combining Hunter glossitis and stomatodynia may be revelatory and lead to early diagnosis of vitamin B12 deficiency. We here report the case of a female patient who had had these oral signs for 2 years and whose laboratory tests revealed normocytic anemia with iron and vitamin B12 deficiency. The diagnosis of vitamin B12 deficiency due to long-term metformin was suspected while excluding other potential causes. Intramuscular hydroxocobalamin injection associated with oral iron led to the normalization of serum levels and to the disappearance of clinical signs. The patient was referred to a specialized center for further etiological assessment. This clinical case highlights the essential role of dentists in early diagnosis of vitamin B12 deficiency and the prevention of its progression, which can be dramatic in the case of late discovery.

## Introduction

Les manifestations buccales d´une carence en vitamine B12 peuvent constituer un motif de consultation chez le dentiste. Une meilleure connaissance de la symptomatologie stomatologique liée à cette carence permet de faire un diagnostic précoce. L´installation de signes hématologiques et surtout neuropsychiatriques constitue une étape tardive de la maladie pouvant laisser des séquelles irréversibles [1]. Les patients sous biguanides constituent une population à risque de carence en vitamine B12 [2]. La surveillance des taux sériques de cette vitamine n´est pas systématique chez ces patients. Les signes buccaux peuvent constituer un signal d´alerte assez précoce permettant de pallier à cette absence de surveillance.

## Patient et observation

**Figure 1:**
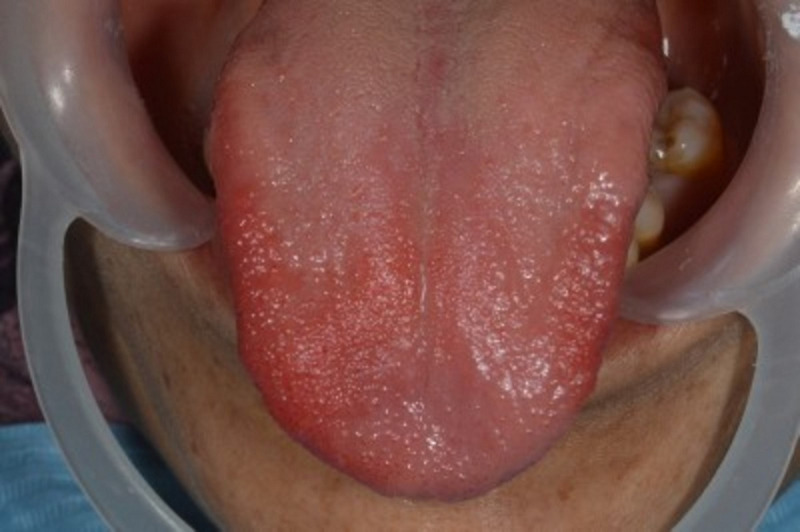
vue supéro-antérieure montrant l'atteinte de la face dorsale de la langue

**Figure 2:**
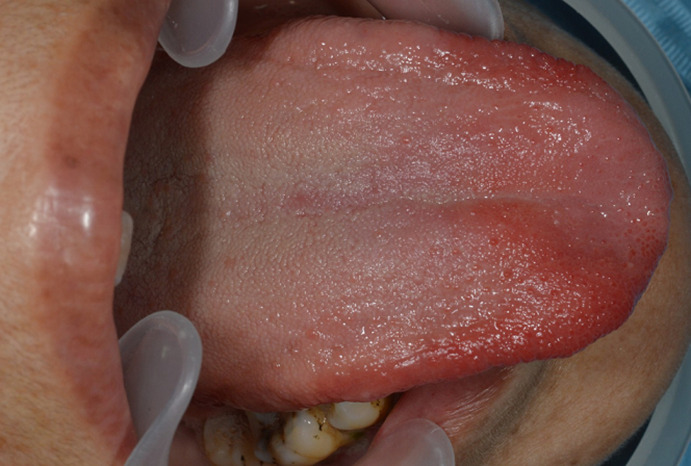
vue supéro-latérale montrant l'atteinte du bord de la langue

Une femme marocaine de 65 ans a été reçue dans notre cabinet pour une algie linguale chronique évoluant depuis 2 ans. Ces douleurs sont à type de brulure empêchant une bonne alimentation et une hygiène orale correcte. Les traitements institués par d´autres médecins dentistes consistaient essentiellement en une association d´antibiotiques (spiramycine et métronidazole), d´antifongiques ainsi que d´antiseptiques locaux type chlorhexidine ou des bicarbonates en bain de bouche. Ces traitements n´ont permis aucune amélioration notable. L´examen endobuccal a révélé la présence d´une parodontite chronique à un stade avancé et une hygiène orale médiocre. Le tiers antérieur de la langue présente sur les faces dorsale et latérales jusqu´à la pointe une zone atrophique, érythémateuse et bien limitée qui semblait migratrice selon les renseignements de la patiente (Figure 1, Figure 2). L´interrogatoire a révélé que la patiente était diabétique depuis 30 ans et qu´elle utilisait, en plus de l´insuline, le metformine à la dose de 2000mg par jour fractionnée en deux prises et ce depuis 15 ans. En plus des signes buccaux, la patiente présente d´autres symptômes neuropsychiatriques: une insomnie, une irritabilité avec tendance dépressive, une perte d´appétit et une asthénie. La patiente s´expliquait la dégradation de son état psychologique par la période de deuil qu´elle traverse (décès de son conjoint depuis 18 mois). Sur le plan neurologique, la patiente présente des vertiges intermittents même en position allongée, des picotements et des décharges électriques aux extrémités supérieures et des fourmillements aux extrémités inférieures.

Devant ce tableau clinique, une avitaminose B12 a été suspectée et un bilan biologique a été demandé comprenant une numération formule sanguine et un dosage de la vitamine B12 et de l´acide folique sériques. Plusieurs anomalies ont été observées: hémoglobine à 10,30 g/100ml (N: 12 à 16g/100ml); hématocrite à 32,90% (N: 35 à 47%); teneur corpusculaire moyenne en hémoglobine (TCMH) à 25,80pg (N: 27 à 33pg); le volume globulaire moyen (VGM) était normal : 82,5Âµ^3^ (N: 80 à 95 Âµ^3^). La vitamine B12 sérique était très diminuée: 80ng/l (N: 180 à 914ng/l). La vitamine B9 était dans les normes. Les fourchettes de « normalité » indiquées sont celles proposées par le laboratoire ayant réalisé le bilan. D´après ces résultats et vu la normalité du VGM et la diminution de la TCMH (ainsi que de l´hémoglobine et de l´hématocrite), une carence martiale a été suspectée. Un dosage de la ferritine sérique a confirmé ce doute: 5,62ng/ml (N: 25 à 280ng/ml) En concertation avec le diabétologue traitant ainsi qu´avec un gastro-entérologue, le traitement antidiabétique est maintenu inchangé et la prise en charge de l´anémie a été instituée par un apport de vitamine B12 en intramusculaire (hydroxocobalmine 5000Âµg avec une injection tous les 5 jours) et du fer per os (fer protéinsuccinylate 40mg à raison de 2 prises journalières). L´utilisation prolongée à grande dose du metformine a été retenue comme étiologie provisoire et plausible de la déficience en vitamine B12 en attendant que la patiente se décide à faire d´autres explorations pour éliminer d´autres causes probables. La patiente a été revue en consultation après deux semaines où l´on a noté une disparition complète de la glossite de Hunter ainsi que de la stomatodynie. Les autres symptômes se sont aussi estompés de façon significative.

## Discussion

Le diagnostic et la prise en charge de cette patiente dont le signe évocateur était stomatologique ont permis de résoudre aussi bien ses problèmes buccaux ainsi qu´une amélioration de ses problèmes neuropsychiatriques. Cette prise en charge a permis de juguler l´évolution de la maladie. La vitamine B12 est un élément essentiel à la synthèse de l´ADN et son déficit retentit sur le turn-over cellulaire ce qui se traduit par des lésions au niveau des tissus à renouvellement rapide comme la muqueuse digestive et en particulier la muqueuse buccale [1,3]. Elle participe également à la synthèse de la myéline ce qui explique les signes neurologiques associés [1]. Il s´agit d´un élément hydrosoluble de structure chimique proche de l´hème avec un atome central de cobalt d´où le nom de cobalamine [4]. Il est fourni exclusivement par l´alimentation d´origine animale. Les besoins quotidiens estimés sont entre 2 et 5Âµg par jour. L´excédent est stocké au niveau du foie. La réserve hépatique varie entre 2 et 5mg ce qui représente 1000 jours d´apport [4]. D´après les données épidémiologiques disponibles, plusieurs travaux ont montré un lien entre une baisse de la vitamine B12 sérique et la prise prolongée du metformine [2,5,6]. Cette baisse est d´autant plus significative que la dose est grande [2,6]. Sur le plan physiopathologique, le principal mécanisme impliqué semble être une maldigestion des cobalamines alimentaires [5].

L´installation de l´anémie avec un syndrome neurologique (paresthésie des extrémités, ataxie, aréflexie tendineuse, sclérose amyotrophique de la moelle) parfois irréversible constitue probablement le signe cardinal du diagnostic de la carence en vitamine B12 [1,5]. Or ce tableau met plusieurs années pour s´installer et il est souvent précédé par des signes cliniques en rapport avec l´atteinte épithéliale. Les lésions buccales sont fréquemment observées avant l´apparition de l´anémie [1], il s´agit classiquement de la glossite de Hunter qui se présente sous deux aspects cliniques: i) une phase inflammatoire ou pré-atrophique caractérisée par la perte de l´aspect velouté de la langue et l´apparition de zones vernissées et de plaques érythémateuses brillantes et sèches, intéressant la pointe et les bords de la langue. Ces zones peuvent parfois s´ulcérer; ii) une phase atrophique d´emblée ou faisant suite à la première avec une langue dépapillée, lisse et de couleur allant du rose pâle au rouge carminé. A la protraction, la langue parait parfois amincie et pointue [4]. L´atteinte linguale complète est rarement rencontrée car elle correspond à des lésions évoluées. Les atteintes partielles de la langue sont les plus fréquemment décrites dans la littérature [1,4]. D´autres lésions de la sphère oro-faciale sont possibles comme les faces internes des joues, la lèvre inférieure, le palais mou ou même le pharynx. Un diagnostic différentiel doit se faire surtout avec une candidose (présence d´un érythème lingual chez les personnes âgées) ou une glossite exfoliatrice migratrice s´il y a migration des lésions comme dans notre cas [1].

Les biguanides dont le metformine peuvent être associés à des déficits et à des carences en vitamine B12 - voire les générer- chez les patients diabétiques [5]. Sur le plan épidémiologique, la fréquence de cette association est de 5,8% à 52% [2]. Le dosage de vitamine B12 n´étant pas systématique chez les diabétiques sous biguanides au long court, l´examen de la cavité buccale peut être un élément de surveillance clinique direct et facile à explorer. La présence d´une glossite (même partielle) et une stomatodynie doivent alerter le clinicien à suspecter une avitaminose B12 [1]. Ces signes précoces - même non spécifiques- vont permettre de juguler une évolution dramatique avec des séquelles neurologiques. Un traitement substitutif avant ce stade permet la disparition totale des symptômes [1,5,7]. Une telle surveillance est facilement réalisable par les médecins dentistes omnipraticiens même en exercice libéral en ville, il suffit juste d´une sensibilisation puisque les lésions sont facilement identifiables [8]. Ceci est vrai pour toutes les carences en vitamine B12 et non seulement celles associées aux biguanides. La présence de signes buccaux évocateurs d´une carence en vitamine B12 doit inciter le praticien à réaliser un bilan biologique comprenant un hémogramme et un dosage de la vitamine B12 sérique [9]. La carence en vitamine B12 est définie en général par une valeur inférieure à 200pg/ml avec une zone grise entre 200 et 300pg/ml [7]. Or, cette définition ne fait pas l´unanimité. En cas de doute, la présence de signes cliniques en faveur de cette carence ainsi que le dosage sérique de l´homocystéine totale ainsi que de l´acide méthyl-malonique permettent de conforter le diagnostic en cas de doute [4].

## Conclusion

Le cas clinique présenté montre le rôle important que peut jouer le médecin dentiste dans le diagnostic des anémies par carence en vitamine B12 ainsi que dans la surveillance de ces carences comme effet indésirable des biguanides (pharmacovigilance). La reconnaissance des lésions buccales associées à ces avitaminoses, bien que facilement identifiables, doit cependant faire l´objet de formation ciblée des médecins dentistes pour éviter l´errance médicale des patients qui en sont atteints et rendre efficace la surveillance de ce risque chez les diabétiques sous metformine.
